# AN69ST membranes adsorb nafamostat mesylate and affect the management of anticoagulant therapy: a retrospective study

**DOI:** 10.1186/s40560-017-0244-x

**Published:** 2017-07-18

**Authors:** Takahiro Hirayama, Nobuyuki Nosaka, Yasumasa Okawa, Soichiro Ushio, Yoshihisa Kitamura, Toshiaki Sendo, Toyomu Ugawa, Atsunori Nakao

**Affiliations:** 1 Department of Clinical Engineering, Okayama University Hospital, 2-5-1 Shikata-cho, Kita-ku, Okayama city, 700-8558 Japan; 2 Department of Emergency and Critical Care Medicine, Okayama University Graduate School of Medicine, Dentistry and Pharmaceutical Sciences, Okayama University Hospital, 2-5-1 Shikata-cho, Kita-ku, Okayama city, 700-8558 Japan; 3 Department of Pediatrics, Okayama University Hospital, 2-5-1 Shikata-cho, Kita-ku, Okayama city, 700-8558 Japan; 4 Department of Pharmacy, Okayama University Hospital, 2-5-1 Shikata-cho, Kita-ku, Okayama city, 700-8558 Japan; 5 Department of Pediatrics, Division of Infectious Diseases and Immunology, Cedars-Sinai Medical Center, 8700 Beverly Blvd., Los Angeles, CA 90048 USA

**Keywords:** AN69ST, Nafamostat mesylate, Continuous renal replacement therapy, Adsorption, Anticoagulant therapy

## Abstract

**Background:**

In Japan, nafamostat mesylate (NM) is frequently used as an anticoagulant during continuous renal replacement therapy (CRRT). The dialyzer membrane AN69ST has been reported to adsorb NM and affect the management of anticoagulant therapy. However, the adsorbed amount has not yet been quantitatively assessed. Therefore, in this study, we evaluated the pre- and post-hemofilter prolongation of the activated clotting time (ACT) in patients with AN69ST and PS membranes. We also measured the adsorption of NM in three types of CRRT membranes using an experimental model.

**Methods:**

In a study of patients who underwent CRRT using AN69ST or PS membranes in 2015 at the Advanced Emergency and Critical Care Center, Okayama University Hospital, pre- and post-hemofilter ACT measurements were extracted retrospectively, and the difference was calculated. In addition, AN69ST (sepXiris100), PS (HEMOFEEL SHG-1.0), and PMMA membranes (HEMOFEEL CH-1.0N) were used in an in vitro model of a dialysis circuit, and the concentrations of NM were measured in pre- and post-hemofilter membranes and filtrates.

**Results:**

The ACT difference was significantly lower in the group using AN69ST membranes (*p* < 0.01). In the in vitro model (*n* = 4) with adsorption and filtration, the post-hemofilter and filtrate concentrations of NM in AN69ST membranes were significantly lower than those in the PS and PMMA membranes (*p* < 0.01). The NM adsorption clearance of the AN69ST membrane was significantly higher than that of the PS and PMMA membranes.

**Conclusions:**

The AN69ST membrane had higher NM adsorption than the PS and PMMA membranes. This may have resulted in the lower ACT difference in patients undergoing CRRT using the AN69ST membrane than in patients undergoing CRRT using PS or PMMA membranes.

## Background

Continuous renal replacement therapy (CRRT) has a milder impact on hemodynamics than intermittent hemodialysis therapy and enables strict management of the body fluid balance, acid-base equilibrium, electrolytes, and plasma osmotic pressure. Accordingly, CRRT has been widely used in the treatment of acute renal injury [1]. However, CRRT requires long-term anticoagulation management [2]. In Japan, nafamostat mesylate (NM) is used in most patients receiving CRRT because it does not affect the patient’s coagulability in vivo [3]. NM is a serine protease inhibitor with a molecular weight of 539 Da that exerts non-antithrombin III-mediated anticoagulant effects by strongly inhibiting activated coagulation factors, such as factor IIa (thrombin), factor Xa, and factor XIIa. In addition, NM also has an inhibitory effect on platelet coagulability. In the blood, NM is rapidly degraded by carboxylesterase; as a result, its active half-life in the blood (β-phase) is 23.1 min. NM can also be eliminated through dialysis. Therefore, NM exerts anticoagulant effects only in extracorporeal circulation circuits, whereas in vivo, NM is quickly inactivated, enabling safe management of coagulation [4].

The AN69ST membrane is currently attracting attention in the field of intensive care because of its ability to adsorb cytokines, making it potentially beneficial in the treatment of patients with septic shock [5, 6]. The AN69ST membrane was developed based on the AN69 membrane, which was released in the market in France in 1969. Because the AN69 membrane has a strong negative charge, it poses problems such as induction of bradykinin production [7] and adsorption of pharmacological agents such as NM [8]. Therefore, with the AN69ST membrane, surface treatment has been performed to neutralize the negative charge at the surface of the membrane. Additionally, reduction of bradykinin production has been achieved by reducing the zeta potential at the point of contact between the blood and the surface of the membrane to a level that is lower than that found in the AN69 membrane [9]. However, few studies have examined the adsorption of drugs by the AN69ST membrane, and no previous studies have quantitatively assessed the amount of adsorption.

Therefore, in this study, the pre- and post-hemofilter difference in the activated clotting time (ACT) in patients using the AN69ST membrane was calculated, and the values were compared with those in patients who used PS membranes. In addition, the amounts of NM adsorbed on AN69ST, PS, and PMMA membranes were quantitatively measured and compared in an in vitro model, and the capacity of the AN69ST membrane to adsorb NM was evaluated.

## Methods

### Measurement of the ACT and calculation of its difference

**Fig. 1:**
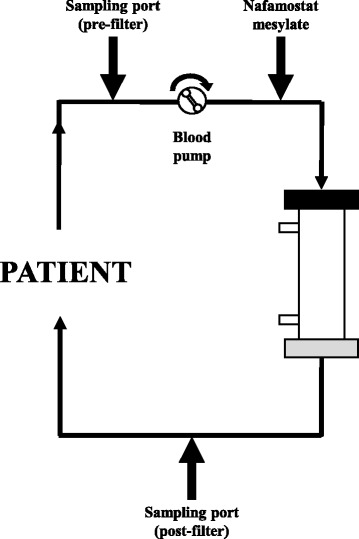
Activating clotting time (ACT) sampling sites

This study was performed in patients who were hospitalized at the Advanced Emergency and Critical Care Center, Okayama University Hospital in 2015, and who were receiving CRRT with AN69ST or PS membranes. In addition, we extracted data for patients whose respective pre- and post-hemofilter ACT was measured simultaneously after initiation of CRRT, and we calculated the ACT difference (ACT difference [s] = ACT post-hemofilter − ACT pre-hemofilter). For the measurement of the ACT, we used a Hemochron Response device (Heiwa Bussan, Co. Ltd., Tokyo, Japan) and Hemochron ACT tubes (Celite ACT; FTCA510; Heiwa Bussan, Co. Ltd.; Fig. 1). This study was approved by the Ethics Committee of Okayama University Hospital (approval no. Ken1610-510).

### In vitro model of a dialysis circuit and measurement of NM levels

The following hemofilters were used (*n* = 4 each): AN69ST membrane (sepXiris100; Baxter Co. Ltd., Tokyo, Japan), PS membrane (Hemofeel SHG1.0; Toray Medical Co., Ltd., Tokyo, Japan), and PMMA membrane (Hemofeel CH1.0N; Toray Medical Co., Ltd.; Table 1). The device used in this study was a JUN-505 (Junken Medical, Co., Ltd., Tokyo, Japan). Only normal saline solution was used as the filling solution to eliminate the influence of other substances. In the clinical setting, heparin coating was performed by adding heparin sodium to the filling solution; however, because the presence or absence of heparin has been reported to not result in any differences in the amount of adsorption of high-mobility group box-1 [10], heparin priming was not performed in our study.

**Table 1:** Characteristics of the three hemofilters

Dialysis membrane	Material	Structure	Surface area (m^2^)	Inner diameter (μm)	Wall thickness (μm)	Priming volume (mL)
sepXiris100	Polyacrylonitrile (surface treated)	Symmetric	1.0	240	50	69
Hemofeel SHG-1.0	Polysulfone	Asymmetric	1.0	200	40	67
Hemofeel CH-1.0N	Polymethylmethacrylate	Symmetric	1.0	200	30	58

**Fig. 2:**
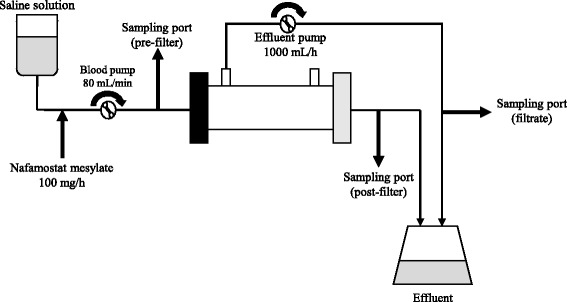
Diagram of in vitro experimental setup (single pass). The heights of all sites of measurement of the inlet pressure, filtration pressure, and return pressure were aligned with the heights of the sites of connection between the dialyzer and the patient. Adjustments were made to maintain a fixed/constant pressure inside the circuit. During sampling, caution was taken not to apply negative pressure. Extracorporeal ultrafiltration method (ECUM) model

To calculate the NM clearance, pre- and post-hemofilter samples and filtrates were collected under the following conditions: blood pump, 80 mL/min; filtrate pump, 1000 mL/h; NM, 100 mg/h (Fig. 2). Although pre-hemofilter samples were collected from the site before administering NM in the clinical setting to observe excess or shortage of anticoagulant, we collected pre-hemofilter samples from the site after administering NM in an in vitro setting to quantify NM adsorption by each membrane. To normalize the sample concentrations, the timing of blood sampling was aligned with that of the time elapsed since the activation of the pump. The timing for sampling was considered as the time when the NM had fully reached the sampling port, which was determined based on measurement of the priming volume of the blood circuit.

The NM used in this study was nafamostat mesylate (MEEK; Meiji Seika Pharma Co. Ltd, Tokyo, Japan), and the normal saline solution used in this study was Terumo (Terumo Corp., Tokyo, Japan). Nafamostat mesylate (Wako Pure Chemical Industries, Ltd., Tokyo, Japan) was used as the standard substance, and ethyl p-hydroxybenzoate (Wako Pure Chemical Industries, Ltd) was used as the internal standard substance.

NM was quantified by high-performance liquid chromatography (HPLC). Measurement devices from Shimadzu Emit Co., Ltd. were used (pump: LC-20AT; ultraviolet/visible detector: SPD-20A; column oven: CTO-20AC; degasser: DGU-20A5R). The HPLC conditions were as follows: flow rate, 1.0 mL/min; wavelength, 260 nm; injection volume, 100 μL; C18-Supersphar column (4 μm, 125 mm × 4 mm); and column temperature, 40 °C. The mobile phase consisted of 0.1 M acetic acid (sodium 1-heptanesulfonate 6.07 g/L):acetonitrile (70:30). An isocratic analysis was performed.

Various equations were used for calculation of NM clearance (Table 2). In addition, for the AN69ST, PS, and PMMA membranes, the sieving coefficient (SC) was 1.0.

**Table 2:** Clearance formulas

Solution clearance (mL/min)	CL_s_ = (CB_i_ − CB_o_)/CB_i_ × (QB − QF) + QF
Filtrate clearance (mL/min)	CL_f_ = CF/CB_i_ × QF
Adsorption clearance (mL/min)	CL_ad_ = CL_s_ − CL_f_
Sieving coefficient	SC = 2CF/(C_Bi_ + C_Bo_)

*C*_*Bi*_ inlet (pre-hemofilter) solute concentration, *C*_*Bo*_ outlet (post-hemofilter) solute concentration, *CF* filtrate solute concentration, *QB* blood flow, *QF* filtration flow

### Statistical analysis

All data are expressed as the mean ± standard deviation (SD) and were compared using chi-squared tests, Mann-Whitney *U* tests, or one-way analysis of variance followed by Tukey’s post-test where appropriate. *p* < 0.05 was considered significant. All statistical calculations were performed using GraphPad Prism 6.0 (GraphPad Software, San Diego, CA, USA).

## Results

### The difference in ACT was significantly lower with the AN69ST membrane

**Fig. 3:**
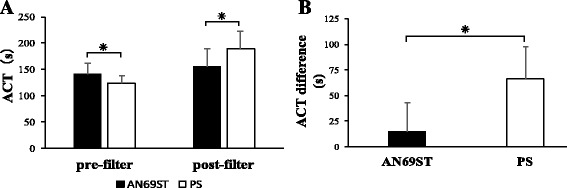
Comparison of ACT difference. **a** Pre- and post-filter ACT for the AN69ST (*n* = 122) and PS (*n* = 37) hemofilters. **b** Pre- and post-hemofilter ACT difference. Data represent the mean (SD) of four independent experiments. *Asterisk* indicates *p* < 0.01 by Mann-Whitney *U* test

There were 122 and 37 ACT measurements in 19 AN69ST-treated patients and 11 PS-treated patients, respectively. Table 3 shows the characteristics of the patients participating in this study. The proportion of sepsis patients was significantly higher in the AN69ST group. Figure 3a shows the pre- and post-filter average ACT in each membrane. Compared with the PS group, the AN69ST group had higher and lower pre- and post-filter ACT values, respectively (*p* < 0.01). Figure 3b shows the pre- and post-filter ACT difference in the AN69ST and PS membrane groups. The ACT difference was significantly lower in the AN69ST membrane group (*p* < 0.01). The amounts of NM used with the AN69ST and PS membranes were 16.8 ± 7.1 and 17.7 ± 8.4 mg/h, respectively, and this difference was not significant.

**Table 3:** Patient summary

Dialysis membrane	AN69ST	PS	*P* value
Age (years)	69.8 ± 11.4	67.4 ± 19.6	N.S.
Sex (male:female)	13:6	6:5	N.S.
Body weight	58.2 ± 14.6	62.3 ± 16.9	N.S.
SOFA	11.1 ± 4.0	9.5 ± 4.4	N.S.
APACHE II	27.6 ± 10.0	26.9 ± 10.4	N.S.
Mortality	6/19 (32%)	3/11 (27%)	N.S.
Sepsis	13/19 (68%)	3/11 (27%)	0.03
QB (mL/min)	95.9 ± 8.9	95.1 ± 15.4	N.S.
QD (mL/h)	934.8 ± 329.6	1422.4 ± 602.5	<0.01
QS (mL/h)	517.7 ± 152.5	602.6 ± 312.8	N.S.
QF (mL/h)	1471.7 ± 414.7	1852.4 ± 662.2	<0.01

Data are the mean and standard deviation or number. For intergroup testing, the Mann-Whitney *U* test or the *χ* ^2^ test was performed at suitable locations. *AN69ST* polyacrylonitrile (surface treated), *PS* polysulfone, *N.S*. not significant, *SOFA* Sequential Organ Failure Assessment, *APACHE II* Acute Physiology and Chronic Health Evaluation II score, *QB* blood pump flow rate, *QD* dialysate pump flow rate, *QF* filtration pump flow rate, *QS* substitution fluid pump flow rate

### NM adsorption was significantly higher with the AN69ST membrane

**Fig. 4:**
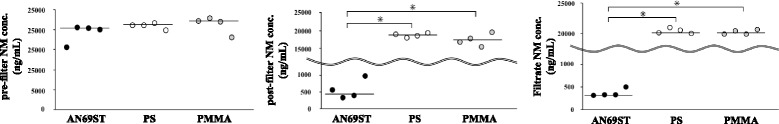
Results of the in vitro assay (single pass). *AN69ST* polyacrylonitrile (surface treated), *PS* polysulfone, *PMMA* polymethylmethacrylate, *conc.* concentration. Study of the adsorbed/filtered amounts of NM (ECUM). Data represent the mean (SD) of four independent experiments. *Asterisk* indicates Tukey’s post-tests that were used to determine the significant differences between groups

**Fig. 5:**
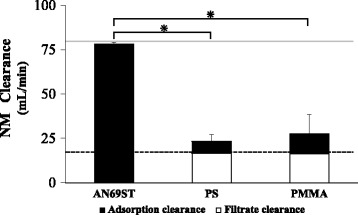
Nafamostat mesylate (NM) clearance. Data represent the mean (SD of total clearance) of four independent experiments. *Asterisk* indicates Tukey’s post-tests that were used to determine differences between the groups. The theoretical maximum value of the filtration clearance is shown as a *dotted line*, and the theoretical maximum value of the adsorption clearance is shown as a *gray line. AN69ST* polyacrylonitrile (surface treated), *PS* polysulfone, *PMMA* polymethylmethacrylate

Figure 4 shows the NM concentrations in pre- and post-hemofilter samples and in filtrate samples when both the blood pump and filtrate pump were activated. The concentrations of NM in the filtrate and post-hemofilter samples were significantly lower in the circuit using the AN69ST membrane than in circuits using the two other membranes (*p* < 0.05; Fig. 4). Finally, we calculated the clearance of NM with each type of hemofilter (Fig. 5). The highest clearance of NM was found when the AN69ST membrane was used (*p* < 0.01); in comparison with the findings obtained with the other membranes, the adsorption clearance (CL_ad_) accounted for a significantly larger proportion, whereas the filtration clearance (CL_f_) did not contribute substantially (*p* < 0.01). Comparable CL_f_ values were found with the PS and PMMA membranes, whereas the CL_ad_ tended to be higher with the PMMA membrane.

## Discussion

AN69 membranes are copolymers of acrylonitrile and sodium methallyl sulfonate and are characterized by their strong negative charge. As a result, positively charged cytokines and NM are adsorbed into the hemofilter through ion binding. For generation of AN69ST membranes, AN69 membranes were subjected to surface treatment with biocompatible polyethyleneimine, resulting in a weaker negative charge [11]. In the clinical setting, heparin priming is generally performed, where the membrane surface is coated with heparin [12]; thus, the negative charge is attenuated by addition of positive charges to the membrane. However, because some negative charges still remain, NM is adsorbed.

The basic principles of substance removal by hemofilters involve the following three elements: dialysis (diffusion), filtration (convection), and adsorption. Given that there is no NM adsorption, diffusion would be the main removal mechanism because NM is small with a molecular weight of 539 Da. Diffusion is correlated with the ratio between the blood pump flow rate (QB) and dialysate pump flow rate (QD). In this study, however, we found a significantly smaller ACT difference between pre- and post-AN69ST hemofilter application, despite the significantly lower QD, indicating low diffusion capability. This finding suggested that NM was removed by adsorption to the AN69ST membrane.

The substance removal capability of each hemofilter can be calculated using the clearance equation. In the model that we used in our experiment, the dialysate was not used to eliminate the effects of solutes other than NM. Therefore, the elimination of NM was examined as a phenomenon that was due to filtration and adsorption. CL_ad_ is defined by the structure and charge of the membrane; the interactions among the charge, half-life, and size of the target substance; and the blood pump flow rate QB. Accordingly, the value of QB is considered to be the maximum value for CL_ad_ [13]. Our experiment was performed with a QB of 80 mL/min; therefore, the maximum value of CL_ad_ was 80 mL/min. In our experimental results, the CL_ad_ of the AN69ST membrane reached a level equivalent to the theoretical maximum value, showing the strength of the membrane’s adsorptive power with regard to NM. CL_f_ represents the product of the filtration pump flow rate (QF) and the sieving coefficient (SC), and the SC is the percentage of solutes that pass through the pores of the hemofilter as a result of the filtration of substances. The three types of hemofilters that we used in our study had a maximal SC value of nearly 1.0 for NM (539.58 Da); therefore, CL_f_ = QF × 1.0, and theoretically, the maximum value of CL_f_ was equal to the QF value [14, 15]. The experiments conducted in our study were performed with a QF of 1000 mL/h, i.e., 17 mL/min. The CL_f_ of the AN69ST membrane was extremely low because most of the NM was lost through adsorption (CL_f_ = 0.3 mL/min). Additionally, for both the PS and PMMA membranes, the CL_f_ reached the theoretical maximum value, showing that these membranes had high filtration capacity in the filtration of NM. The PMMA membrane had a neutral to weakly negative charge and had the ability to adsorb substances in the pores inside the membrane. As a result, the CL_ad_ of the PMMA membrane tended to be higher than that of the PS membrane.

There are some limitations in this study. First, the patient sample size was small. The proportions of men differed greatly; however, there were no significant differences between groups, possibly because of the small sample size. Second, the proportion of patients with sepsis was significantly higher in the AN69ST group. Sepsis-associated factors may have affected the NM adsorption ability of the AN69ST filter. Third, further studies are necessary to determine how the adsorption capacity of the circuit model used in our study may change over time. Additionally, only NM was measured in this study. Accordingly, measurements were performed using normal saline solution as the filling solution. In the clinical setting, various substances and drugs in the blood are also believed to be adsorbed. Therefore, NM concentrations may need to be measured from samples collected from dialysis circuits attached to actual patients.

## Conclusions

In summary, a comparison of pre- and post-hemofilter findings revealed that in dialysis patients, the ACT difference was significantly lower with the AN69ST membrane than with the PS membrane. Moreover, our study showed that this was due to the high NM adsorption capacity of the AN69ST membrane. We suggest that in dialysis circuits using AN69ST membranes, administration of additional post-hemofilter doses of NM may be useful for the management of anticoagulant therapy.

## Abbreviations

ACT: Activated clotting time
AN69: Polyacrylonitrile
AN69ST: Polyacrylonitrile (surface treated)
CL_ad_: Adsorption clearance
CL_f_: Filtration clearance
CRRT: Continuous renal replacement therapy
ECUM: Extracorporeal ultrafiltration method
NM: Nafamostat mesylate
PMMA: Polymethylmethacrylate
PS: Polysulfone
QB: Blood pump flow rate
QD: Dialysate pump flow rate
QF: Filtration pump flow rate
QS: Substitution fluid pump flow rate
SC: Sieving coefficient
SDs: Standard deviations
